# Topic and Trend Analysis of Weibo Discussions About COVID-19 Medications Before and After China’s Exit from the Zero-COVID Policy: Retrospective Infoveillance Study

**DOI:** 10.2196/48789

**Published:** 2023-10-27

**Authors:** Duo Lan, Wujiong Ren, Ke Ni, Yicheng Zhu

**Affiliations:** 1 School of Digital Media and Design Arts Beijing University of Posts and Telecommunications Beijing China; 2 School of Journalism and Communication Beijing Normal University Beijing China; 3 New Media Research Center Beijing Normal University Beijing China

**Keywords:** zero-COVID policy, topic modeling, Weibo, COVID-19 medications, social risk, personal risk, social media, COVID-19, China, pandemic, self-medication

## Abstract

**Background:**

After 3 years of its zero-COVID policy, China lifted its stringent pandemic control measures with the announcement of the 10 new measures on December 7, 2022. Existing estimates suggest 90%-97% of the total population was infected during December. This change created a massive demand for COVID-19 medications and treatments, either modern medicines or traditional Chinese medicine (TCM).

**Objective:**

This study aimed to explore (1) how China’s exit from the zero-COVID policy impacted media and the public’s attention to COVID-19 medications; (2) how social COVID-19 medication discussions were related to existing model estimates of daily cases during that period; (3) what the diversified themes and topics were and how they changed and developed from November 1 to December 31, 2022; and (4) which topics about COVID-19 medications were focused on by mainstream and self-media accounts during the exit. The answers to these questions could help us better understand the consequences of exit strategies and explore the utilities of Sina Weibo data for future infoveillance studies.

**Methods:**

Using a scrapper for data retrieval and the structural topic modeling (STM) algorithm for analysis, this study built 3 topic models (all data, before a policy change, and after a policy change) of relevant discussions on the Chinese social media platform Weibo. We compared topic distributions against existing estimates of daily cases and between models before and after the change. We also compared proportions of weibos published by mainstream versus self-media accounts over time on different topics.

**Results:**

We found that Weibo discussions shifted sharply from concerns of social risks (case tracking, governmental regulations, etc) to those of personal risks (symptoms, purchases, etc) surrounding COVID-19 infection after the exit from the zero-COVID policy. Weibo topics of “symptom sharing” and “purchase and shortage” of modern medicines correlated more strongly with existing susceptible-exposed-infected-recovered (SEIR) model estimates compared to “TCM formulae” and other topics. During the exit, mainstream accounts showed efforts to specifically engage in topics related to worldwide pandemic control policy comparison and regulations about import and reimbursement of medications.

**Conclusions:**

The exit from the zero-COVID policy in China was accompanied by a sudden increase in social media discussions about COVID-19 medications, the demand for which substantially increased after the exit. A large proportion of Weibo discussions were emotional and expressed increased risk concerns over medication shortage, unavailability, and delay in delivery. Topic keywords showed that self-medication was sometimes practiced alone or with unprofessional help from others, while mainstream accounts also tried to provide certain medication instructions. Of the 16 topics identified in all 3 STM models, only “symptom sharing” and “purchase and shortage” showed a considerable correlation with SEIR model estimates of daily cases. Future studies could consider topic exploration before conducting predictive infoveillance analysis, even with narrowly defined search criteria with Weibo data.

## Introduction

### Background

China is one of the few countries that has sustained a stringent zero-COVID policy for almost 3 years [1,2]. It was hoped that the policy would suppress the infection rate to low levels. Its realization was characterized by regional quarantines, regular (sometimes daily) polymerase chain reaction (PCR) tests, and the categorization of households, communities, and neighborhoods into high-, mid-, and low-risk ones.

In November 2022, Chinese authorities started to ease the zero-COVID policy, with “新20条” (“20 new measures”), which only superficially changed the zero-COVID policy, with easing of quarantine time and interprovincial PCR checkpoints and simplification of the categorization of high-, mid-, and low-risk zones. The termination of the zero-COVID policy occurred on December 7, 2022, when the “新10条” (“10 new measures”) became effective. This eradicated almost all restrictions on social mobility and regulated PCR tests in most of China [3].

The lifting of the zero-COVID policy was expected by epidemiologists to be one of the most consequential policy changes about COVID-19 control in China [4,5]. Meanwhile, the end of the zero-COVID policy also led to a gradual discontinuation of official case reports. Using regional survey data and partial PCR and antigen test data for calibration, 1 estimation showed China might have witnessed an unprecedented amount of daily new cases during December 2022, potentially with an epidemic-doubling time of 1.6 days [6]. It is estimated that 90%-97% of the whole Chinese population was infected in the same month [6].

### Social Media Data for COVID-19 Infoveillance

Infoveillance is a comparatively new methodological approach in medical informatics research. It is tightly connected to the subdomain of infodemiology, which is “the science of distribution and determinants of information in an electronic medium, specifically the Internet, or in a population, with the ultimate aim to inform public health and public policy” [7]. Infoveillance is an important approach to study infodemiological phenomena. More specifically, it stands for “the longitudinal tracking of infodemiology metrics for surveillance and trend analysis” [8].

Numerous studies have used social media as an infoveillance tool for COVID-19 outbreaks to understand information content and communication opportunities. In public health crisis scenarios, publics would engage in health communication behaviors on social media. First, health care authorities and professionals can communicate with the general public on social media about the best practices of COVID-19 infection prevention [9]. Second, research has studied factors influencing how publics learn and communicate about COVID-19 vaccines online [10]. Third, publics also engage in discussions about potentially effective treatments and medications for COVID-19 [11,12].

Moreover, infoveillance studies can help public health policy makers understand epidemic trends more efficiently, while also providing reasonable accuracy. Yet, social media infoveillance usually faces challenges stemming from the complexity and sensitivity of topic scope and choices [7]. For the case of China, Shen et al [13] found that some, but not all, topics (ie, topic about symptoms) correlate with official case counts [13]. The quantity of the posts on symptoms has a significant Granger causality relationship with official report cases with varying time lags. Although official case count data were challenged by susceptible-exposed-infected-recovered (SEIR) model estimation [6], social media data can be an important reference for the actual trend of infection during the exit from the zero-COVID policy. This study illustrates the possibility to use targeted keywords for infoveillance using Sina Weibo data.

Nonetheless, existing research also has not paid much attention to social media trends after reopenings or lifting of lockdowns. One study showed that the United States reopening/lifting the lockdown was met with increased positive sentiments on social media. However, they eventually declined with rising fears of catching the virus [14]. However, China’s exit from the zero-COVID policy, as discussed earlier, could be drastically different to reopening policies in the United States. For one thing, the medications and health resources in reserve per capita could be much less in China [15].

In addition, few studies have examined medication-related topics. Existing research on social media discussions about lockdown lifting or reopenings has largely focused on the politicized or polarized nature of policy debates in the Western context [16,17]. The more important question for Chinese social media users, and those in the Global South [18], was perhaps not about whether to reopen but about how to prepare themselves for the imminent infection waves. In China, treatments for COVID-19 depend on either modern medications or traditional Chinese medicine (TCM). China’s massive population also challenges the medication and health care availability during a short exit window.

To bridge these gaps in the literature and to explore the unique characteristics of social media discussions after China’s exit from the zero-COVID policy, this study aimed to contrast Chinese social media trends about COVID-19 medications with existing estimates of case counts. It also aimed to reveal publics’ attention to different COVID-19 medication topics before and after the exit. We present a retrospective infoveillance effort to meet these goals. The initial steps illustrate topic distribution and the correlation between trends. Hence, we raised the following research questions (RQs):

- RQ1: What were the major topics from November 1 to December 31, 2022?
- RQ2: How well do different topical trends correlate with estimations of daily cases?

### Personal vs Social Risk Perceptions

From a health communication perspective, studies have found that Chinese publics use Weibo, the Chinese equivalent to Twitter, as a platform to gain COVID-19 knowledge, seek help, or express distinct kinds of emotions [19]. Topics and themes shifted as policy, epidemic, or seasonal factors came and faded [20]. This shows that Weibo’s topics about COVID-19 can be used to understand publics’ shifting concerns of COVID-19 issues.

The ending of the zero-COVID policy is likely to change online attention to different types of health risks [14]. In this study, we distinguished between topics about personal versus social risks. This dichotomy is an important theoretical framework in health communication [21]. We aimed to apply such dichotomy to the illustration of topic differences related to COVID-19 medication and treatments before and after the exit.

Personal versus social risk perceptions are concepts related to each other. The personal risk perception involves oneself or one’s close social circles (friends, family, etc), and the social risk perception involves other more remote individuals in society [22]. Lifting of lockdowns and monitoring programs could increase the (perceived) chances of infection for the general population [14]. Although psychobehavioral mechanisms are conditioned by many other factors, the termination of the zero-COVID policy or lockdowns can change the relative weight of these perceptions and therefore people’s social media discussions [23,24].

In earlier studies [21,22], personal risk perception was associated with interpersonal communication channels, while its societal counterpart was associated with mass media. However, social media platforms create channels at both the personal level (online friends and circles) and at the societal level (official accounts, online communities, etc). Therefore, existing research shows that social media discussions about a health crisis often include topics related to both types of risk perceptions [21].

### Topics About Personal vs Social Risk of COVID-19 Medication on Social Media

Empirically, the framework of personal versus social risk perception was used to explain topical differences and trends in online health communication. During lockdowns, publics perceived that COVID-19 infection posed a risk more to others in society than to themselves [25]. Members of communities with high collectivist values engaged in mutual help to provide medication and treatment support [26]. However, after reopening or lifting of lockdowns, social media discussions about the fear of infection increased in the United States [14].

More specifically, this paper explores whether the end of the zero-COVID policy switched public perceptions of social versus personal risk [22]. Studies have found that before the lifting of lockdowns in China, social media discussions were concentrated on alerts of new emergent cases and societal management strategies [27]. Based on the hypothesis of optimistic bias, at such a stage, publics are arguably more concerned with societal levels of risks rather than personal levels of risks [25]. Numerous studies have shown that this is true for not only general health communication scenarios but also COVID-19 crises [28,29]. In other words, low infection rates brought by lockdowns or social control methods would keep personal risk perception lower than social risk perception.

After previous liftings of lockdown measures in other countries, social media discussions about COVID-19 death tolls, symptoms, treatments, and medications increased in the United States, United Kingdom, and Iran [14,17,18]. Based on psychometric models of health and crisis communication, existing studies have found publics’ increased perception of their level of involvement to be an important factor influencing such communicative behaviors [30]. As personal involvement with COVID-19 was perceived or expected to be high, people would become increasingly concerned about treatment and medication information and resources [31,32].

In other societal settings, research has found that cancellation of lockdown policies usually led to new waves of COVID-19 infections [17,33-35] and would thus change how citizens perceive their level of involvement in the COVID-19 risks. Therefore, their attention toward COVID-19 vaccines and medications increased [36,37]. In some studies, the lifting of lockdown policies was found to have increased misinformation about COVID-19 treatment on social media or was related to panic-buying or stockpiling of medication resources [38-41].

As China exited from the zero-COVID policy, the risk of infection became ultimately personal and real. Topics surrounding COVID-19 medications and treatments on social media could be a reflection of Chinese publics’ concerns in response to policy change. Based on these considerations, it is expected that they would shift their attention to certain topics surrounding COVID-19 treatment and medication, while setting other topics aside. Hence, we raised the following RQ and hypothesis:

- RQ3: What were the most prominent topics before and after the 10 new measures?
- Hypothesis 1 (H1): Proportionally, there will be more weibos about personal risks (symptoms, shortage, etc) after the 10 new measures.

### Mainstream and Self-Media Accounts on Chinese Social Media

Weibo is one of the oldest and largest social media platforms in China and also an important arena where information about COVID-19 is circulated and discussed [42,43]. Social media platforms, such as Weibo, Twitter, and Facebook, are often used to share users’ views, emotions, and experiences [44]. As of the first quarter of 2022, Weibo had 582 million users, making it the social media platform with the largest user base within China [45]. According to data from September 2020, among users of Weibo, the proportion of male users was 45.4%, while the proportion of female users was 54.6%. These users predominantly consist of individuals born after 1990, accounting for approximately 80% of the total [46]. In addition, approximately 68% of Weibo users have attained a bachelor’s degree or a higher educational qualification [47]. To a certain extent, the user base of Weibo can represent the main viewpoints and opinions of the current Chinese population.

Weibo is a platform where mainstream accounts influence and interact directly with ordinary online publics (also known as self-media accounts) [48]. It provides a labeling function that distinguishes blue-label accounts (official or governmental agencies, mainstream media) from red-label accounts (people or organizations with verified identities) and nonlabel accounts (other accounts) [49]. Given such a structure, studies have compared how official discourse, narratives, and topical focus differ from those of the rest of the Weibo accounts (self-media accounts) [50].

More specifically, the effect of intermedia agenda setting on controversial issues was also found on Weibo between official accounts and other accounts [51]. Scholars have illustrated that official and ordinary accounts can have significant differences in terms of their focus on agendas of COVID-19 news [52]. In certain scenarios, such as public information sharing, mainstream accounts predominantly influence the agenda and discussions about new cases, tracking information, etc [43,53]. However, during crisis times, such as the initial outbreak of COVID-19 in Wuhan, self-media accounts on Weibo and other social media took a more leading position in information sharing and mutual assistance [19,54].

In addition to social media’s benefits for health and risk communication, these platforms can also facilitate the distribution of misinformation, especially about medications and treatments for COVID-19 [55]. Chinese mainstream accounts are predominantly state owned or state supported and play an important role in combating online rumors and providing authoritative informational guidance [48]. One of the expected functions of mainstream accounts on Weibo is to combat rumors and misinformation about COVID-19, including fake therapies and counterfeit medications [56]. Moreover, research has found that Chinese mainstream accounts are concerned with public opinions about COVID-19 policies and would thus intervene in the agenda-building process of controversial topics, when necessary [50].

Presumably, given the positioning of mainstream accounts on Chinese social media, it is expected that they would shift agendas as the general publics shift their attention to personal risks [51]. Their focus on certain COVID-19 medication topics can reflect authorities’ attention to public opinion trends. Hence, we raised the following RQ4: Proportionally, what are the preferential differences between mainstream and self-media accounts on the topics surrounding COVID-19 medication (1) before and (2) after the 10 new measures?

## Methods

### Data Collection

In terms of structure and functionality, Weibo is similar to Twitter. This study collected Weibo data using a Python-based web scrapper. The time frame was set from November 1 to December 31, 2022, putting the time of the announcement of the 10 new measures roughly in the middle of the whole time frame (December 7).

We focused on discussions involving COVID-19 medications among Weibo users using a specific keyword search strategy. First, “药” (“medicine”) became the central keyword in relevant discussions. In addition, homophonic memes have become an important way of expressing implicit meanings on Chinese social media platforms. They are used when explicit expressions are undesired or not allowed [57,58]. The diagnosis of COVID-19 infection is referred to as having been tested positive. In the Chinese context, “positive” (“阳”) has the same pronunciation as “sheep”/“goat” (“羊”).

Before we proceeded with data collection, we obtained 200 randomized Weibo posts using the search term “新冠” (“COVID-19”) as the keyword. We then manually screened each post to see whether it was a discussion about COVID-19 medication, and 166 (83%) of them were suitable for analysis. Further, we counted the text word frequencies of the 166 posts. The results showed that 74 (44.6%) of the posts were characterized by “阳” (“positive”), 85 (51.2%) by “羊” (homophonic of “positive”), and 160 (96.3%) by “药” (“medicine”). No other words accounted for more than 20% of the posts. To a certain extent, it can be said that words that accounted for more than 40% of the posts were able to cover most of the Weibo posts on COVID-19 medications.

**Figure 1:**
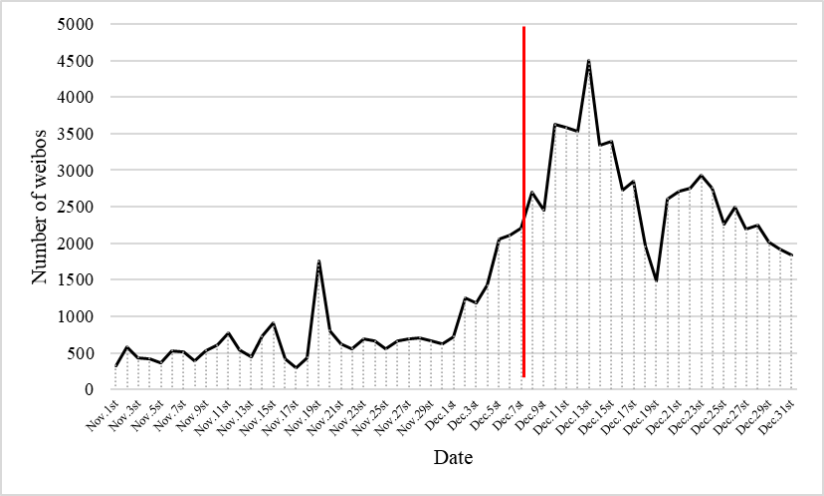
Trend of weibos relating to COVID-19 medications from November 1 to December 31, 2022, in China (N=94,082 weibos from 62,720 Weibo accounts, including mainstream and self-media accounts). The red line indicates December 7, 2022, the date when the 10 new measures ended the zero-COVID policy in China.

To ensure scope fit while maximizing inclusivity, we used 3 keyword combinations for data collection first, including “新冠” (“COVID-19”) or “阳” (“positive”) or “羊” (homophonic of “positive”) + “药”(“medicine”). The scrapper targeted data labels of “publish_time,” “user_name,” “content,” and “verify_type” of each weibo. After the data-cleaning process, we were able to obtain a data set containing 94,082 weibos from 62,720 Weibo accounts. The trend of all weibos can be seen in Figure 1.

### Analytical Strategy

#### Structural Topic Modeling and Time Frames

In general, this study was interested in the effect of time and Weibo account types on the distribution and proportion of topics about COVID-19 medications and treatments in China. To operationalize time periods, we chose December 7, 2022, as the cut-off time point and split the whole time frame into 2 parts: “before a policy change” (November 1-December 6) and “after a policy change” (December 7-31) periods. We use the structural topic modeling (STM) package in R (R Core Team and the R Foundation for Statistical Computing) for the topic modeling procedure [59]. Next, 3 separate models were selected, respectively, for the whole time frame (model 1), before a policy change (model 2), and after a policy change (model 3) [60]. See Table 1.

**Table 1:** Weibo models related to COVID-19 medications before and after a policy change.

Models, time frames, and account types			Accounts, n (%)		Weibos, n (%)
**Model 1 (whole time frame: November 1-December 31, 2022)**					
	Mainstream	6006/62,720 (9.6)		21,619/94,082 (23.0)	
	Self-media	56,714/62,720 (90.4)		72,463/94,082 (77.0)	
**Model 2 (before 10 new measures: November 1-December 6, 2022)**					
	Mainstream	2732/16,975 (16.1)		7377/27,024 (27.3)	
	Self-media	14,243/16,975 (83.9)		19,647/27,024 (72.7)	
**Model 3 (after 10 new measures: December 7-31, 2022)**					
	Mainstream	5003/49,891 (10.0)		14,242/67,058 (21.2)	
	Self-media	44,888/49,891 (90.0)		52,816/67,058 (78.8)	

#### STM’s Robustness and Accuracy Judgments

This study ensured the robustness and effectiveness of topic modeling in 2 aspects. First, STM provides optimal topic number exploration analysis. In other words, by comparing the relevance and interpretability of outputted topic words within a specified range of topic numbers—model package parameter k, such as k=c(3:20)—the best balance between exclusivity and semantic consistency in the number of topics can be determined. Second, STM constructs a network for running the model on the given topic number (refer to the model manual), sequentially calculating the model’s relevant parameters. The program continues running until the model converges or reaches the maximum iteration limit, resulting in a scatter plot that includes 2 variables: semantic coherence and exclusivity. The scatter plot is then used to select an appropriate model for further analysis (see Multimedia Appendix 1).

We repeated these 2 steps for the 3 models in sequence. In addition, in the selection of topics, we did not solely rely on the program’s output results. Two trained coders independently manually coded the recommended topic numbers from the program to ensure the identification of the most suitable number of topics. The manual coding judgments were based on the following criteria: clear and coherent semantics within each topic and semantic distinctiveness between topics.

The results of semantic consistency and perplexity indicated that model 1 fit 6 or 11 topic numbers, model 2 fit 5 or 9 topic numbers, and model 3 fit 6 or 8 topic numbers. Manual judgment determined that there were 11 potential topics for model 1, 9 potential topics for model 2, and 8 potential topics for model 3. We further determined the optimal model parameter configurations for the 3 models. These correlation analyses and their parameters are visualized in Multimedia Appendix 1.

#### STM Topics and Themes

Quantitatively, the STM algorithm estimates proportions of topics within each model. For STM, each weibo had different probabilities to “fall under” a certain topic among all topics. The number of topics (k) was determined using diagnosis of semantic coherence and exclusivity of the weibos under each topic (which is a reiterative procedure, as illustrated in Multimedia Appendix 1). The coherence and exclusivity indexes help researchers maximize the convergent and discriminant validity of topics as measurements [59]. Aggregately, STM can estimate the proportion of weibos a topic would take at a given time or during a period of time. STM does not definitively say whether a weibo belongs to a certain topic, so the topics can be considered as largely but not absolutely mutually exclusive. However, the expected proportions can be a reliable reflection of the relative topic strengths [59]. STM has also been used in other social media content analyses surrounding COVID-19 topics [61,62].

Qualitatively, each model then provides a suggested list of topics and the keywords that characterize its content. After cross-referencing the keywords with raw data using STM’s *findThoughts* function, each topic was qualitatively assigned a summary title by reverse-checking relevant keyword-containing weibos. In each model, we further categorized topics into meaningful sets of themes, which are larger summarizations of topics. Themes are more generic and exist consistently within the whole time frame and can thus be qualitatively compared across models.

#### Account Types

As mentioned earlier, we also collected the account type data on each Weibo post and categorized all the accounts in our data set as either mainstream account or self-media account. Such categorization is based on the account verification provided by Weibo and a list of government- and authority-affiliated media accounts. In previous studies, Weibo accounts that are not blue-verified have often been referred to as self-media accounts [63]. We followed this concept and used it to refer to all microblog accounts except mainstream media and official organizations. We filtered the blue-verified accounts to obtain only verified media accounts and then combined the 2 lists to form a final list of mainstream accounts and self-media accounts. Quantitatively, the STM algorithm also estimates proportions of topics contributed by different types of accounts in sum and across time.

### Ethical Considerations

The abovementioned data are publicly available. No data were used to trace personal identification information, such as ID number, birth date, or IP address. The authors consulted the Institutional Review Board of the residing institution and were informed that no approvals were required.

## Results

### Topic Distribution in the Whole Time Frame (RQ1)

RQ1 was related to the topic distribution and themes that emerged from the data of the whole transition period. The model 1 result from the STM analysis is shown in Table 2. There were 11 potential topics identified in the whole time frame. These topics could be further categorized into 3 general themes: (1) symptoms and treatments, (2) governmental pandemic control policies, and (3) COVID-19 case-tracking information. The first theme focused mainly on personal risks, while the second and third themes focused on social risks. During the whole time frame, “symptoms and treatments” was the most prevalent theme.

**Table 2:** Main themes in weibos (N=97,028) related to COVID-19 medication before and after a policy change.

General themes and subthemes			Example weibo
**Theme 1: symptoms and treatments**			
	Sharing symptoms and medicine purchase experiences	“…heard about the new policies are coming and went out to prepare my medication inventory, after visiting a dozen of pharmacies I finally get all the things on my list.”	
	Suggestions of treatments	“COVID-19 is a self-limiting disease; effective medicines include proprietary traditional Chinese medicines...”	
**Theme 2: governmental pandemic control policy**			
	News about policy change	“Continue to ameliorate pandemic control policies: here are what you need to know about the ten new measures...”	
	New policy implementation	“After the new policies have been announcement, we have examined how they are being implemented on the streets...”	
**Theme 3: COVID-19 case-tracking information**			
	Case information and medicine suggestions	“17 new cases in Beijing today….we need to stick with scientific quarantine measures…Do not purchase medicine or take medicine on your personal efforts...”	
	Trace information sharing	“New Cases in Waning City…case No.74, 75, 76, 78 lives in Huayuan community, and have visited Longxing Hotel and a drug store...”	

As shown in Table 2, these 3 general themes were identified quantitatively to maximize their mutual exclusivity. The first theme “symptoms and treatments” included weibos sharing personal COVID-19 experiences and symptoms around the word “positive” (“阳/羊”). Weibo users associated “positive” with “medicine” (“药”) to describe the difficulty in obtaining medication and to suggest medications and treatment plans based on their personal experience or official guidance. The second theme “governmental pandemic control policies” included discussions about recent policy changes, the purpose of new policies, and how the policies are being implemented. The third theme “COVID-19 case-tracking information” included mainly governmental or mainstream media’s disclosure of new infection data and trace information. These weibos also had the keyword combination of “COVID-19” or “positive” with “medicine” as they were mostly concentrated before the “10 new measures” policy change, suggesting that people “be compliant with quarantine measures and not buy medicine or take medicine personally.”

A detailed distribution of the topics estimated by the STM algorithm is shown in Table 3 (keyword lists are in Multimedia Appendix 2). The most prevalent topics about personal risks surrounding COVID-19 medication were COVID-19 medicine stockpiling, purchase, and delivery (n=21,705, 22.37%, of the total weibos), TCM treatments plans (n=18,697, 19.28%), and symptom sharing (n=17,620, 18.16%). These were followed by the availability of nirmatrelvir/ritonavir, Azulfidine, and other oral antivirus drugs (n=8927, 9.20%), as well as discussion about inhalable vaccines by CanSino (n=9052, 9.33%). Topics about social risks of COVID-19 medication largely concentrated on medication recommendations given out, together with other governmental pandemic control policies and infection-tracking information. In the whole transition period of the zero-COVID policy, Weibo discussions about personal risks surrounding COVID-19 medications were proportionally higher than those surrounding social risks.

**Table 3:** Expected topic distribution of the whole time frame (model 1).

Main theme and topic label			Personal risk proportion (N=97,028), n (%)		Social risk proportion (N=97,028), n (%)
**Theme 1: symptoms and treatments (n=76,001, 78.33%)**					
	3. Purchase and shortage	21,705 (22.37)		—^a^	
	7. TCM^b^ formulae	18,697 (19.28)		—	
	2. Symptom sharing	17,620 (18.16)		—	
	5. Inhalable vaccines	9052 (9.33)		—	
	1. Oral antivirus drugs	8927 (9.20)		—	
**Theme 2: governmental pandemic control policy (n=17,097, 17.62%)**					
	4. Worldwide policy comparison	—		8820 (9.09)	
	9. Governmental investigation	—		4473 (4.61)	
	6. Pandemic control policy	—		3804 (3.92)	
**Theme 3: COVID-19 case-tracking information (n=3930, 4.05%)**					
	8. Cases in Zhengzhou and Dalian	—		1582 (1.63)	
	10. Cases in Wuhan and Beijing	—		1436 (1.48)	
	11. Cases in Xi’an and Xinzhou	—		912 (0.94)	

^a^Not applicable.

^b^TCM: traditional Chinese medicine.

These results show that people’s risk perceptions surrounding COVID-19 medication cannot be limited to the development and effectiveness of medication. In fact, topics showed that people also faced risks of the shortage or unavailability of certain medications (eg, nirmatrelvir/ritonavir, Azulfidine, ibuprofen, Tylenol), as well as uncertainties created by the medication approval process, generic drugs, drug prices, side effects, etc. Risks also originated from the delivery process, and keywords such as “send,” “mail,” “urge,” and “received” were the most used in relevant discussions. These observations highlight the importance of informational preparedness for both authorities and general publics about medications during the policy transition time of the COVID-19 pandemic.

Moreover, Weibo users expressed various types of discrete emotions (ie, fear, anger, disgust) in the discussions. Before a policy change, the “symptom sharing” topic included keywords such as “die” and “afraid.” Potentially, “die” was used metaphorically to express the feeling of extreme discomfort in an exaggerated manner. Past research has shown that emotional thinking is a key antecedent of the consumption of misinformation and rumors. Emotional expressions took a large proportion in the 3 models, and they potentially led to more risks in the decision-making process of Weibo users in terms of information selection, permitting and forfending COVID-19 medication.

### Corroborating Topic Trends With SEIR Estimation (RQ2)

**Figure 2:**
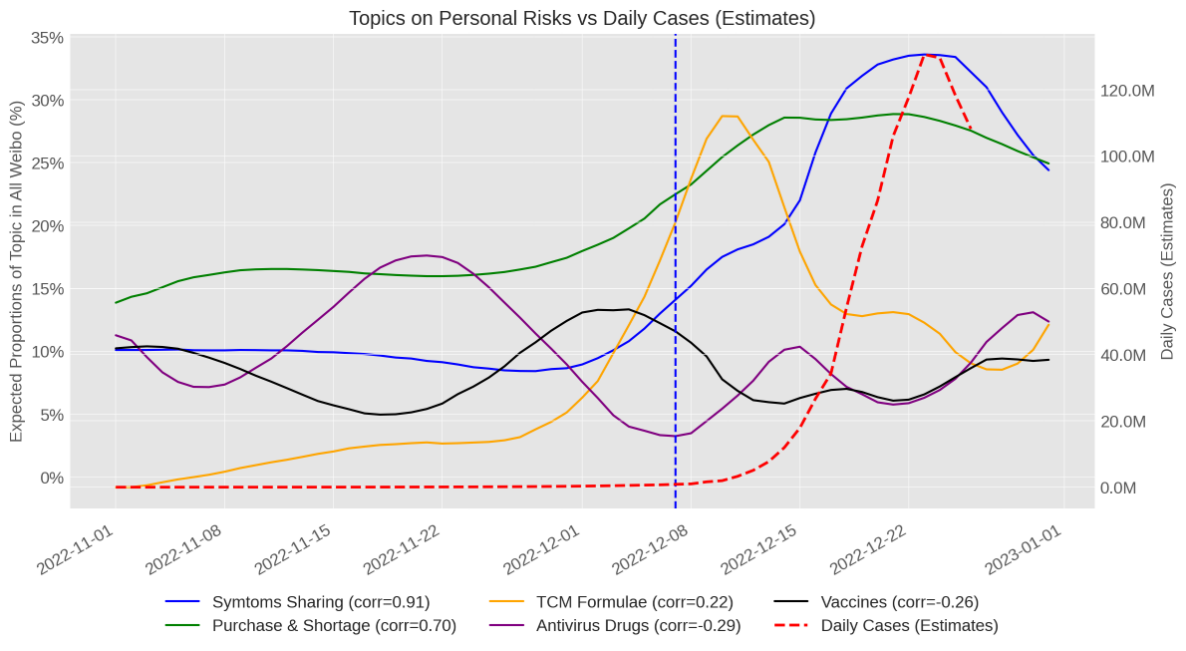
Trends of topics about personal risks compared to case estimates. Time frame: November 1-December 26, 2022. Left y axis: proportions of weibos in the topic relative to all weibos (N=94,082); right y axis: number of daily cases; x axis: dates. The dotted red line shows estimated cases (in millions) by Goldberg et al [6]. The dotted blue line marks the exit date (December 7, 2022) from the zero-COVID policy (10 new measures). Pearson correlation values between Weibo data and estimated cases are shown in legends. TCM: traditional Chinese medicine.

To answer RQ2, the expected proportional changes of topics related to symptoms and treatments were illustrated and contrasted with other topics (Figure 2). Within the theme of symptoms and treatments, the 3 most prominent topics, namely topics 2, 3, and 7 (Figure 2, blue, green, and yellow lines), had a much higher proportion after the date of 10 new measures than before. The other 2 topics, oral antivirus drugs and inhalable vaccines, experienced fluctuations during the whole time frame (Figure 2, purple and black lines). For oral antivirus drugs, the highest proportion was reached around November 20, which included discussions about the approval and availability of Pfizer’s Paxlovid (nirmatrelvir/ritonavir) and Azulfidine. A discussion about the development of CanSino’s inhalable vaccine had its peak around the time of the 10 new measures.

**Figure 3:**
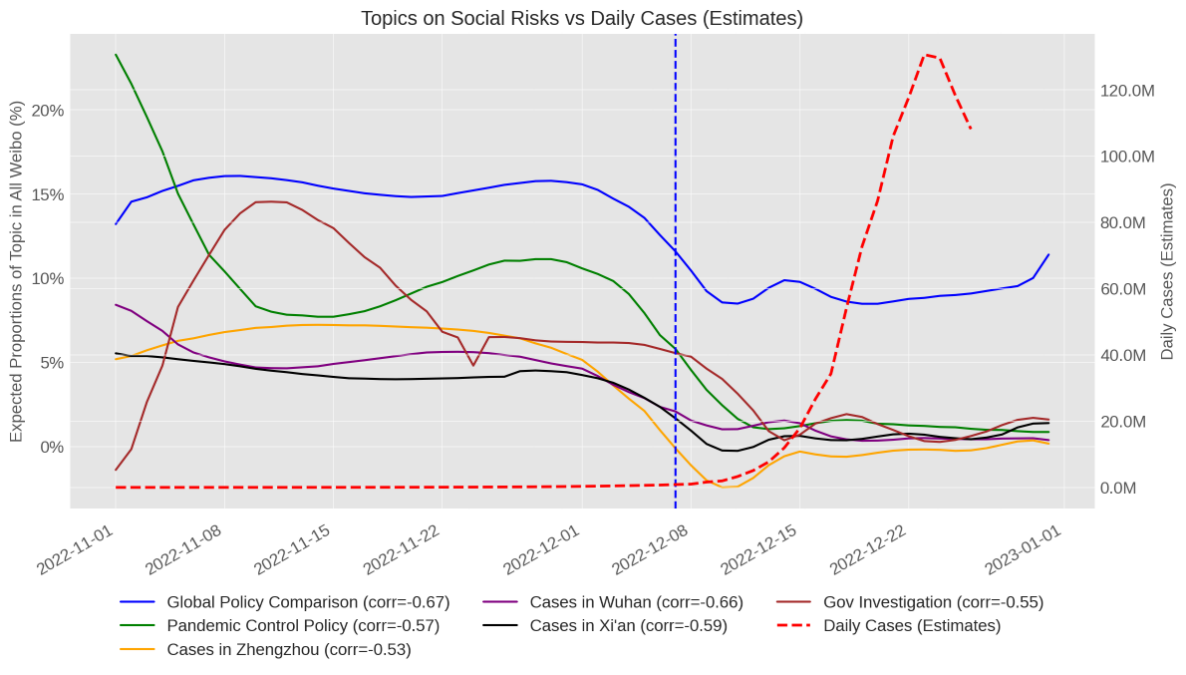
Trends of topics about social risks compared to case estimates. Time frame: November 1-December 26, 2022; left y axis: proportions of weibos in the topic relative to all weibos (N=94,082); right y axis: estimation of daily Cases; x axis: dates. The dotted red line shows estimated cases (in millions) by Goldberg et al [6]. The dotted blue line marks the exit date (December 7, 2022) from the zero-COVID policy (10 new measures). Pearson correlation values between Weibo data and estimated cases are shown in legends.

This study considered themes 2 (governmental pandemic control policies) and 3 (COVID-19 case-tracking information) as social risks. These topics had a higher popularity before the 10 new measures than after. Topics 6, 7, 9, and 10 almost completely faded away (Figure 3).

Official case reports became unreliable after November 11, 2022, and official PCR test records were also incomplete. Due to the absence of reliable official data and other limitations, this study relied on existing estimations of daily cases [6], which is the only estimation to date to the best of our knowledge. Among all topics related to personal risk, symptom sharing had the highest correlation with estimated daily cases (r=0.91), purchase and shortage ranked second (r=0.70), and other topics about personal risks had much lower values. Social risk topics (Figure 3), however, all had negative correlations with estimated daily cases.

### Comparison of Topics Before and After a Policy Change (RQ3 and H1)

**Figure 4:**
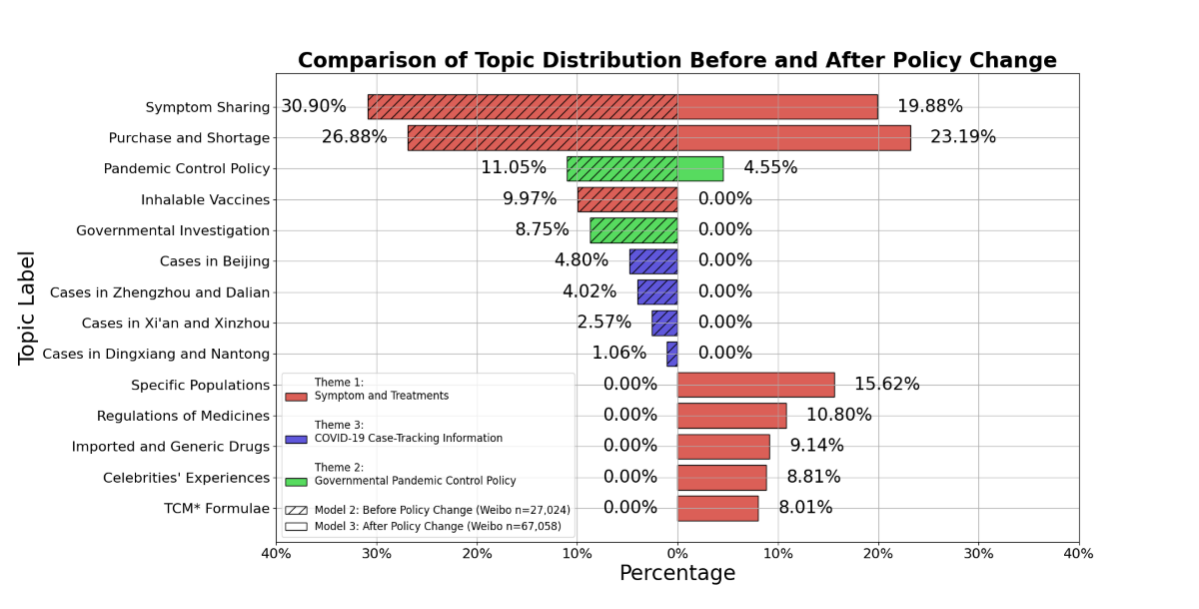
Comparison of topic distribution about COVID-19 medications before and after a policy change (the 10 new measures that ended the zero-COVID policy in China). Topics were estimated separately for structural topic models of model 2 (including weibos before a policy change from November 1 to December 6, 2022; n=27,024) and model 3 (including weibos after a policy change from December 7 to 31, 2022; n=67,058). Y axis: topic labels; x axis: proportions of weibos within each model. Colors represent general themes. Nonshaded bars pertain to model 2, and shaded bars pertain to model 3. TCM: traditional Chinese medicine.

To answer RQ3 and test H1, this study explored 2 separate topic models based, respectively, on data before a policy change (model 2) and after a policy change (model 3). As shown in Figure 4 (keyword lists are in Multimedia Appendix 2), model 2 (before a policy change) included 9 topics pertaining to the same 3 themes identified in model 1 (whole time frame). Model 3 (after a policy change), however, included 8 topics but had only 2 emergent themes relating, respectively, to symptoms and treatments and to governmental pandemic control policies.

First, comparisons between the 3 models (Table 3 and Figure 4) showed that the 3 general themes identified in the whole model can summarize all the topics in the data set. Using these 3 themes as baseline, we found no new topics emerging in models 2 and 3.

The 2 models differed in the proportions of weibos concerning different types of risks (personal vs social). Before a policy change (model 2), the most prevalent topics were symptom sharing (n=8350, 30.90%) and medication purchase and treatment plans (n=7264, 26.88%). Overall, about 57.78% (n=15,614) of all weibos were concerned with personal risks, while the rest were concerned with the social aspects of medication-related services, such as PCR tests or quarantine policy changes. In these “social risk” topics, discussion about COVID-19 medication only appeared as supplementary information in public announcements and was usually led by mainstream accounts rather than self-media accounts.

Topics after a policy change (model 3) were mostly focused on the “symptoms and treatments” theme, with more evenly distributed interests around different aspects of medication. Proportionally, the most prevalent topics were medication purchase and availability (n=15,551, 23.19%), symptom sharing (n=13,331, 19.88%), and treatment plans for children and people with preexisting conditions (n=10,474, 15.62%). In contrast to model 2, the proportion of symptom sharing dropped from 30.90% (n=8350) to 19.88% (n=13,331) in model 3 (2-sample proportion test: z score=36.32, *P*<.001). Although Figure 1 shows that there were more weibos in model 3, this drop in proportion showed that Weibo users became more concerned with other personal risks, such as shortage, import of foreign-made drugs, generic drugs (mainly from India and Bangladesh), and addition of new medicines to the social-security-covered drug list.

In sum, the most prevalent themes in models 2 and 3 were symptoms and treatments (67.75% and 95.45%, respectively). Topics surrounding COVID-19 medications and treatments diverged into more granulated aspects, and “personal risk” topics increased proportionally to 95.45%, while “social risk” topics dropped to 4.55% from 32.25% (χ^2^_1_=13506.16, *P*<.001). This result supports H1. The “symptoms and treatments” theme is the only theme that predominantly expresses (self-media accounts) or addresses (mainstream accounts) personal risks related to COVID-19 medication, including shortage, misusage of medicines, worrying about the elderly and children in the family, and sharing symptoms.

### Mainstream vs Self-Media Accounts Before a Policy Change (RQ4)

**Figure 5:**
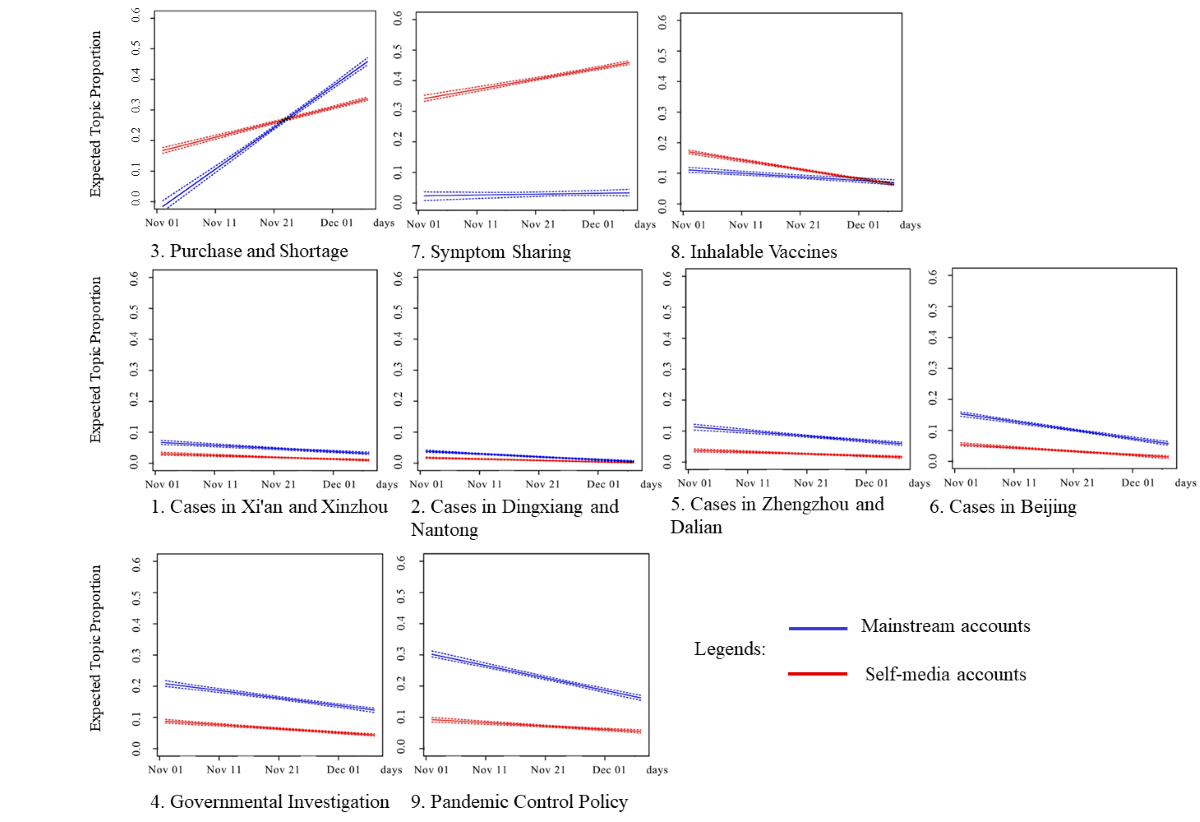
Linear trends in mainstream and self-media accounts’ participation in topics before a policy change. Time frame: November 1-December 6, 2022; y axis: proportions of weibos on the topic relative to all weibos (n=27,024); x axis: time in days. Trends are linear transformations by STM of the changes in weibo percentages from a particular source type (mainstream accounts vs self-media accounts) across the time frame. STM: structural topic modeling.

For each theme and its topics, proportions of weibos from mainstream versus self-media accounts are contrasted across time in Figure 5. For the period before the 10 new measures, more self-media accounts discussed topics under the “symptoms and treatments” theme compared to mainstream accounts. Symptom sharing was dominated by self-media accounts through the whole “before a policy change” period. However, mainstream accounts gradually took over self-media accounts on the topic of availability of medications and treatment plans around November 19, 2022, when online sales of Paxlovid without a prescription was prohibited by authorities. Other topics and themes related to social risks in model 2 (before a policy change) showed gradually declining trends.

### Mainstream vs Self-Media Accounts After a Policy Change (RQ4)

**Figure 6:**
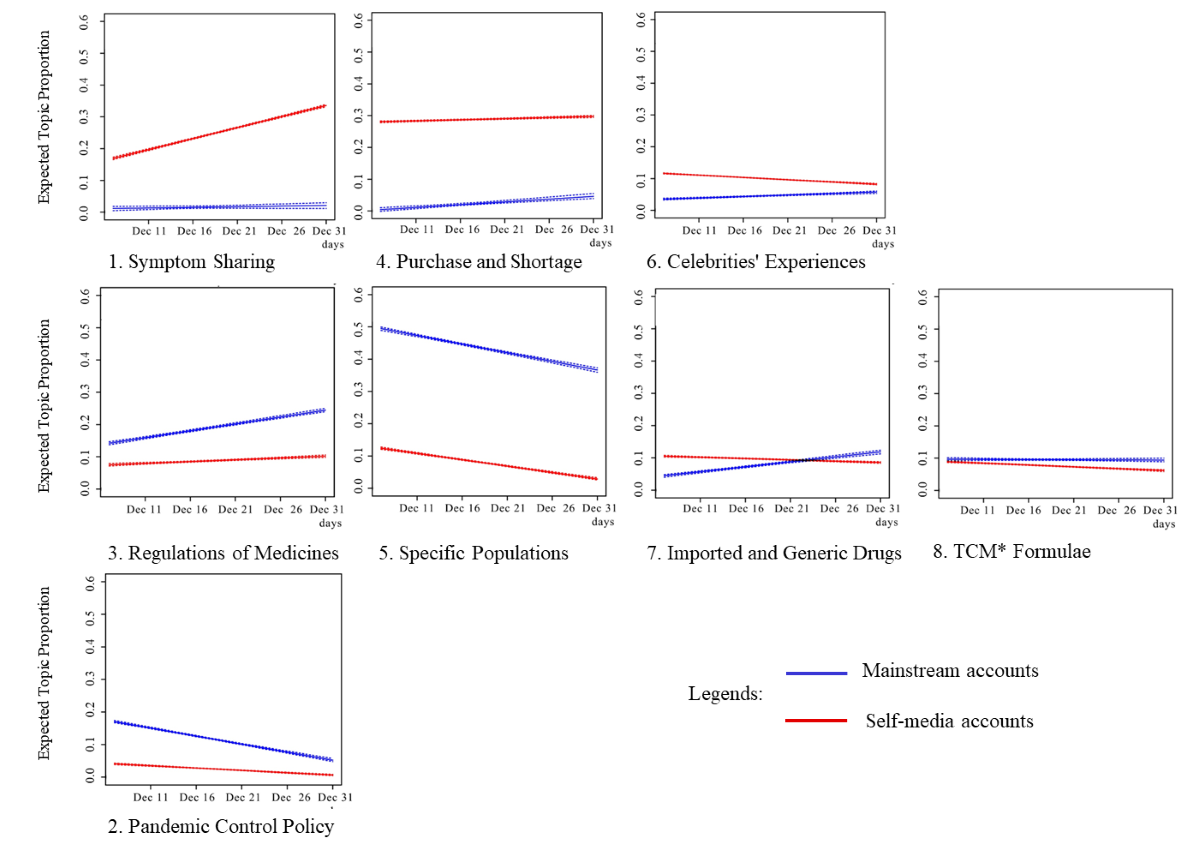
Linear trends in mainstream and self-media accounts’ participation in topics after a policy change. Time frame: December 7-31, 2022; y axis: proportions of weibos on the topic relative to all weibos (n=67,058); x axis: time in days. Trends are linear transformations by STM of the changes in weibo percentages from a particular source type (mainstream accounts vs self-media accounts) across the time frame. STM: structural topic modeling; TCM: traditional Chinese medicine.

Figure 6 illustrates mainstream versus self-media accounts’ discussion about the topics after a policy change (model 3). In comparison to the period before a policy change (model 2), topics and themes in model 3 had a more balanced distribution between the 2 types of accounts. Among the 7 topics related to symptoms and treatments, symptom sharing and medication purchase/shortage were dominated by self-media accounts. Weibos about celebrities’ symptoms constituted a single topic and were also discussed more by self-media accounts.

Mainstream accounts, however, had a higher proportion of topics about approval of medications and officially recommended treatment plans, as well as treatment suggestions and plans for children and people with preexisting conditions. Mainstream accounts also had a higher proportion of the topic about governmental pandemic control policies.

The only topic where mainstream accounts had a lower proportion first but then surpassed self-media accounts is oral antivirus drugs, such as Paxlovid. This topic discussed issues surrounding availability of the drug in different regions of the world, most notably the Chinese mainland, Hong Kong, and the United States.

## Discussion

### Principal Findings

This study confirmed that China’s exit from the zero-COVID policy impacted social media discussions’ attention to COVID-19 medicines, treatment plans, symptoms. In the context of COVID-19 treatment and medications in China, the results showed that the topical trends in “symptom sharing” and “purchase and shortage” better correlated with existing estimates of case counts during the exit. Moreover, discussions increased in volume after the zero-COVID policy was lifted. The quantity of medication-related weibos at its peak was about 5 times that at the beginning of our observation. The increase in such discussion could potentially be attributed to the following reasons: First, the perceived risk of actually becoming infected increased after a policy change; second, there was only limited access to (the information about) antivirus drugs before a policy change, and these medications appeared in a key topic after the 10 new measures; and third, mainstream accounts on Weibo were topic leaders and agenda setters in many medication-related topics before the end of the zero-COVID policy.

### Topic Characteristics During China’s Exit From the Zero-COVID Policy

The results in response to RQ1 showed that social media discussions about COVID-19 included a mixture of governmental instructions on medication, public sharing of treatment plans, and public outcry for medication and health care resources.

Few studies have specifically examined social media topics after the lifting of lockdowns or reopening policies, and their attention to COVID-19 medications was even less [14,24,64,65]. Our result has 1 topic common with an existing study about the United States reopening in 2020 [14]—that social media discussions include emotions such as fear and anxiety toward potential infection (after the second postlockdown phase in the United States). Different to both US and UK reopening studies [14,24], we found that Weibo discussions surrounding COVID-19 medications in China after the zero-COVID policy focused more on seeking medical resources and sharing experiences with medication shortage.

Arguably, this may not be a balanced comparison, as previous reopening studies did not specifically focus on “medications” as a keyword but rather policy names. However, in the UK study, none of the social media topics was connected with medication [24], and for both US and UK studies, social media discussions were to a certain extent politicized and polarized about reopening policies [14,24]. In our Weibo data, we found some topics related to debates about policy choices and comparisons with other reopening strategies (eg, the worldwide policy comparison, trend in Figure 3 and in Multimedia Appendix 2). However, they are not as predominant as in US and UK data sets. Public attention to this topic dropped as the exit happened (and cases started to rise) in China. Potentially, when personal health risk was on the rise, social media users in China made less effort to discuss policies and different versions of exit strategies.

In our Chinese data, we found TCM to be a similarly, if not more, important topic as modern medicines. This shows that topics about COVID-19 medication are embedded in a larger online cultural context [66]. Arguably, in other societal context where traditional medicines are respected and used (eg, Korea, Malaysia, Jordan, India) [67-70], there may appear separate topics about treatment plans or promotions of traditional medicines. With the results presented in the next section, we cannot definitively conclude whether social media topics about them are useful data or noise for infoveillance studies, but we propose that this could be reinspected in future studies.

In the specific context of mainland China, these discussions showed the difficulty in managing the medication requirements of a massive population. From a certain perspective, topics such as symptom sharing and purchasing of medications illustrated the massive need of self-medication. Self-help, mutual aid, and some other spontaneous community measures have proven to be effective in dealing with COVID-19 [58]. Given the difficulty in obtaining timely prescriptions of antivirus drugs and factors such as availability and panic buying, ordinary publics used social media as a platform to search for and share/sell medications, perhaps without formal instructions from doctors [71]. Our results also showed that TCM formulae or proprietary Chinese medicines remained prominent in topics in all 3 models, especially in the whole data set (model 1) and in the later period (model 3). Arguably, treatment plans and medications related to or originated directly from traditional formulae are still highly respected and practiced by both the authorities and ordinary publics in China.

In sum, the results about social media topics can help future infoveillance studies to anticipate the content of medication-related social media discussions. Notably, social media users’ complaints about medication shortage can be a representation of medication needs. Although emotional expressions are found to be an important predictor of social behavior [72], this topical trend can potentially help increase model predictiveness for researchers and identify public interest in social media monitors. This will be further discussed in the next subheading.

### Comparing Trends in Symptoms, Shortage, and TCM With Existing Estimates

The results corresponding to RQ2 showed that online discussions about medication share/exchange market on Weibo can be separated into several temporal trends. In Figure 2, there is a trend line of purchase and shortage, which focused on modern medications, and that of TCM formulae, which focused on TCM. The “purchase and shortage” trend peaked on day 43 (December 23, 2022), while the “TCM formulae” trend peaked earlier, on day 41 (December 11, 2022).

Using a combination of case count (discontinued after December 23, 2022, by the Chinese Centers for Disease Control and Prevention) and online prevalence surveys conducted on December 26, 2022, in China, an existing study estimated in an SEIR model that the Omicron outbreak in China after the exit from the zero-COVID policy peaked around December 23-24, 2022 [6].

In comparison to the trends in symptoms, shortage, and TCM during the whole time frame (Figure 2), existing estimates correlated most with the “symptoms and shortage” trends in the Weibo data in terms of peak time estimates. The “TCM formulae” trend, however, peaked earlier at just 4 days after the ending of the zero-COVID policy. In addition, we found stronger relationships (than the TCM discussions) between the SEIR estimates and discussions about symptoms and shortage of modern medication. The reason that the TCM trend peaked sooner than other trends can be manifold: primarily, specific TCM formulae are consistently recommended by the authorities as official treatment plans and are encouraged in clinical treatment and self-medication [66]. The immediate rising trend in the TCM topic after the exit could be a reflection of official advocacy of TCM use after the exit. Figure 6 also illustrates that mainstream accounts gained a narrow proportional advantage than self-media accounts on the TCM topic after the exit.

This finding could be useful for future infoveillance studies using Weibo data; it recommends that total volumes of weibos could show different trends than SEIR modeling based on real-world data when official data are potentially underreported [6]. Even if this study sets a narrow and stringent search strategy targeting only COVID-19 medications, the whole corpus contains topics with drastically different trends. STM can to a certain extent help social media infoveillance researchers filter important topics.

### Impact of Policy Change on Public Attention to Risks

Our data showed that weibos in all 3 models have clear and generally consistent patterns in themes: symptoms and treatments, governmental pandemic control policies, and COVID-19 infection-tracking information. For the whole time frame, the first general theme was about personal risks of COVID-19 and how medications can or cannot be acquired to address such health risks. The latter 2, pandemic control policies and case tacking, were about social risks. These include governmental policy updates, warnings of regional outbreaks, instructions of how (not) to use certain medications, and how measures are implemented accordingly to cut off transmission routes.

After several countries reopened in 2020, researchers argued that the “repeal and alteration of lockdown policies mark a symbolic transfer of responsibility for epidemic control from state to individual” and that it might catalyze fear for infection [17]. Accordingly, the results related to RQ3 and H1 illustrate the exit’s significant impact on public attention to different types of risks. Between the 20 new measures (alleviated stringent social control on November 11, 2022) and the 10 new measures (abandoned the zero-COVID policy on December 7, 2022), the discussion about COVID-19 medications on Weibo was still dominated by social risks [53]. However, we found that interests in social risks waned as time progressed, especially after the 10 new measures, which completely annulled the zero-COVID policy.

This result shows that China’s exit also shifted publics’ attention to the increasing risks faced by individuals and their personal responsibilities in epidemic prevention. This is similar to the observations on exit or reopening policies in other countries [64,65]. In our case, official announcements are an important section of online discussions less explored by previous studies [14,24,43,64,65,73]. In fact, case tracking was not recognized by the STM algorithm as a separate topic after the 10 new measures. Figure 5 illustrates that case-tracking topics were dominated by mainstream accounts. Most likely, the disappearance of case tracking among topics is a result of the sudden abandonment of case-tracking reports by local authorities soon after the 10 new measures.

### Types of Perceived Personal Risk After a Policy Change

The results of RQ3 also helped identify 3 most prominent types of personal risks of COVID-19 medication in the post-zero-COVID period. The first type is the perceived risk of becoming infected (symptoms). After a policy change, discussions about symptoms and treatments diverged and became more granulated in terms of specific scope.

First, the different trends in Figures 2 and 3 show that people became more concerned about actual consequences and symptom-coping strategies. Before the 10 new measures, COVID-19 medication discussions appeared more in governmental reports about regional cases and were more likely to be recommendations. After the 10 new measures, COVID-19 medications became a necessity for many who became infected.

The limited availability of effective medication constituted the second type of perceived personal risk. First, discussions about the difficulty in purchasing antivirus drugs and delays in delivery of online orders showed that many were anxious about medication access. Second, our results showed that TCM and modern medications are 2 separate topics; it appears that TCM topics did not include expressions of urgency and anxiety. These 2 observations show that Weibo discussions had a heavier emphasis on the unavailability of modern medicines than TCM.

The limited availability of health care resources was the third type of perceived personal risk. First, antivirus drugs were not included in the National Reimbursement Drug List (NRDL) when the zero-COVID policy was lifted. Although the authorities included them into the NRDL near the end of December 2022 in limited regions in China [74], Weibo discussions still showed anxiety about the unavailability of either Chinese or imported antivirus drugs. Pfizer’s Paxlovid was sold at around 3000 Chinese yuan (~US $435) in China [75,76], and the price is around 75% (120%) of the monthly income of urban (rural) Chinese.

### Differences in Issue Construction and Agenda Influence Between Mainstream and Self-Media Accounts

Like in other health crisis scenarios in China, a major part of mainstream accounts on Weibo has the responsibility to manage online communication about issues surrounding COVID outbreaks [77]. Our data showed that mainstream accounts were dominant in topics such as quarantine and outbreak information before a policy change. As people’s need for COVID-19 medication surged, mainstream accounts endeavored to respond with instructional information about medication access, delivery, and usage.

Before a policy change, mainstream accounts were more active only on topics related to regular infection monitoring and governmental pandemic control policies. However, they became more active than self-media accounts on treatment plans after November 20, 2022 (Figure 5, “Purchase and Shortage”), when online sales of unprescribed Paxlovid were temporarily and regionally prohibited and public demand was on the rise [76]. This shows that mainstream accounts responded to the trending discussions about COVID-19 medication shortage and stockpiling before the 10 new measures.

As discussed earlier, the “symptoms and treatments” theme became more granulated. Communication-wise, the expansion of the issue scope might have challenged the responsiveness of mainstream accounts in all topics [53]. As shown in Figure 6, mainstream accounts managed to compete with self-media accounts on “regulation of medicines” and “imported and generic drugs” but could not catch up with the discussion surrounding “shortage and purchase.” This illustrates the key topics that mainstream accounts decided to focus on during a health communication emergency.

### Limitations

In terms of generalizability, certain patterns in topics surrounding COVID-19 medications and treatment in this study may be particularly suitable for the social and cultural context of mainland China, including policy change mechanisms and public culture in drug prescription and usage. Moreover, STM is not able to provide more in-depth data analysis with targeted keywords. Potential keywords such as “sequelae” or “side effect” were not specifically searched for or filtered during data analysis. In addition, due to Weibo’s policy and the difficulty in obtaining individual data, regional and socioeconomic factors were not analyzed in the study.

### Conclusion

This paper describes the online discussions about COVID-19 medications and treatments on the Chinese social media platform Weibo before and after easing of the zero-COVID policy. Results suggest that lifting pandemic control policies will significantly impact publics’ interests in topics about COVID-19 medication and treatment risks in terms of scale and scope.

By contrasting the trends in different topics surrounding COVID-19 medications with existing estimates from SEIR models, the study found that trends in topics about symptoms and drug shortage/purchase correlated with existing estimations about the peak of the outbreak after China’s exit from the zero-COVID policy (around December 23-24, 2022). The TCM topic trends spiked much earlier with a high topic proportion.

Discussions shifted focus from concerns about social risks, such as treatment recommendations after PCR tests and regional outbreaks, to personal risks related to COVID-19 medications and became more specific in terms of the scope of the risks. Mainstream accounts were found to be mostly responsive to publics’ concern about COVID-19 medications and have preferences for policy-related topics, such as regulations about reimbursement and import of foreign drugs.

Future health crisis responders could benefit from anticipating risk concerns related to medications, as well as increasing society’s informational and behavioral preparedness during health policy changes. For infoveillance research in China, the study provides a case where different topics are compared with exiting peak time estimates. It is recommended that social media content used for infoveillance in China consider the complexity of medication traditions in China and the potential difference between trends in modern medicines and TCM during a health emergency.

## Appendix

Multimedia Appendix 1 Diagnosis of topic sematic coherence and exclusivity for the 3 models.

Multimedia Appendix 2 Topic distribution, proportions within models, and keywords.

## Abbreviations

NRDL: National Reimbursement Drug List
PCR: polymerase chain reaction
RQ: research question
SEIR: susceptible-exposed-infected-recovered
STM: structural topic modeling
TCM: traditional Chinese medicine

## Data Availability

The data sets generated and analyzed during the study are available from the corresponding author upon reasonable request. Figures 2 and 3 adopted daily estimated cases from a susceptible-exposed-infected-recovered model [6], and some models were reproduced in the study for comparison using data and codes provided by Goldberg et al [6].
