# Supplementary material for: Topic and Trend Analysis of Weibo Discussions About COVID-19 Medications Before and After China’s Exit from the Zero-COVID Policy: Retrospective Infoveillance Study
Source: J Med Internet Res. 2023 Oct 27;25:e48789. doi: 10.2196/48789 (PMC10638631; doi:10.2196/48789)
Supplement: Multimedia Appendix 1 [file jmir_v25i1e48789_app1.pdf]

Appendix II. Diagnosis of Topic Sematic Coherence and Exclusivity for the Three Models

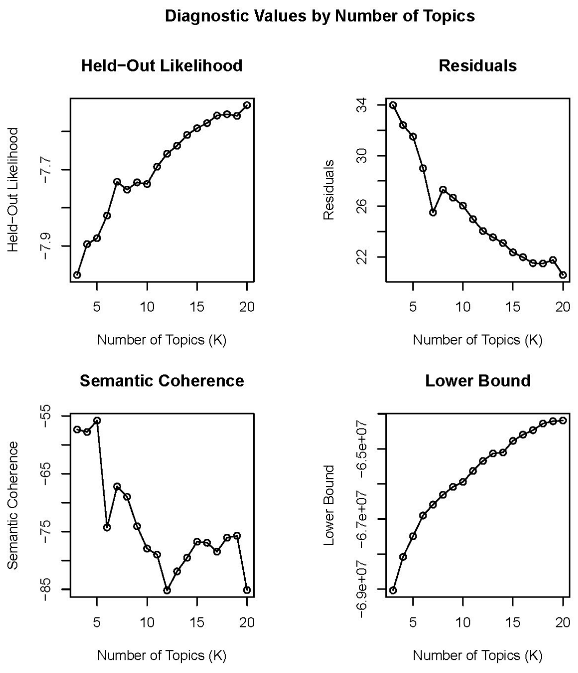

Diagnostic Values for Model 1 (Whole Timeframe)

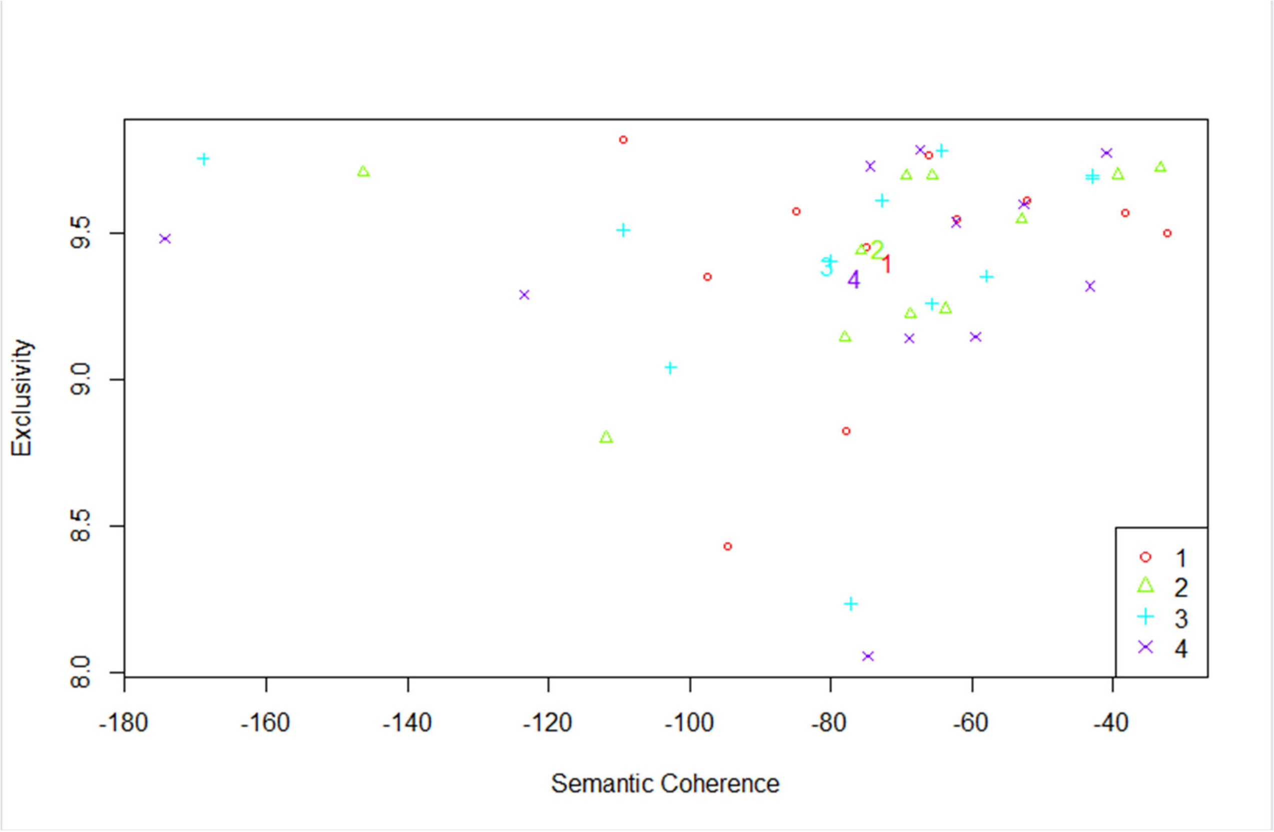

Model 1 Semantic Coherence and Exclusivity Plots.

Colors indicate different model iterations,

Colored numbers indicate averaged values for a color-represented iterations.

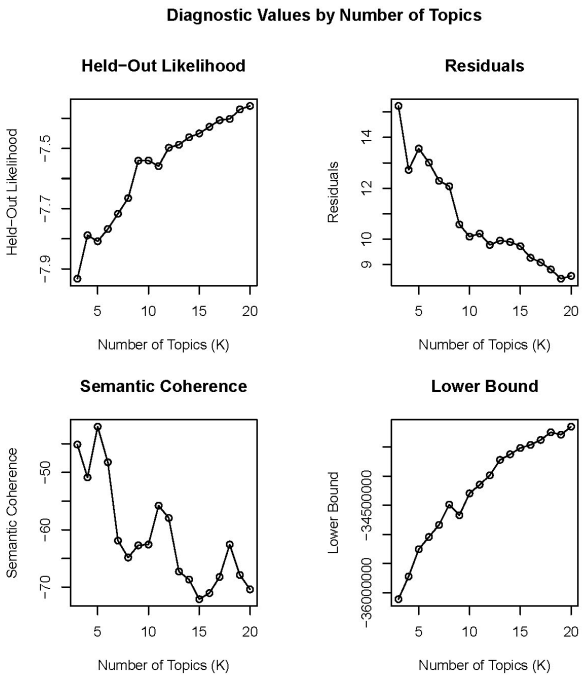

Diagnostic Values for Model 2 (Before Policy Change)

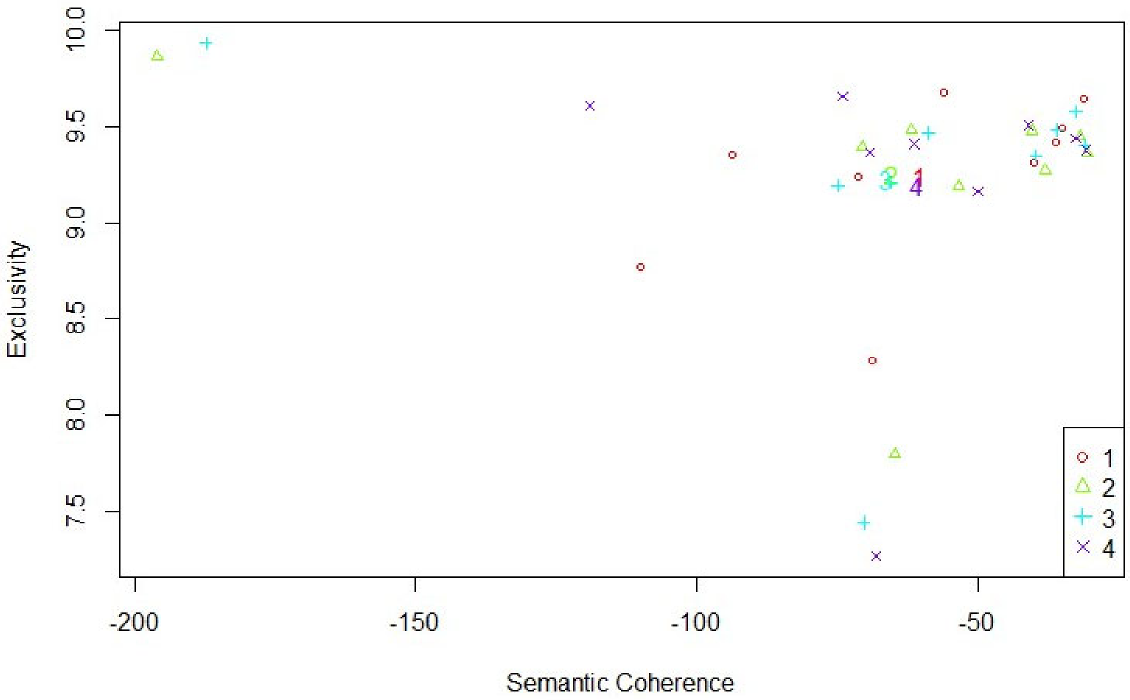

Model 2 Semantic Coherence and Exclusivity Plots.

Colors indicate different model iterations,

Colored numbers indicate averaged values for a color-represented iterations.

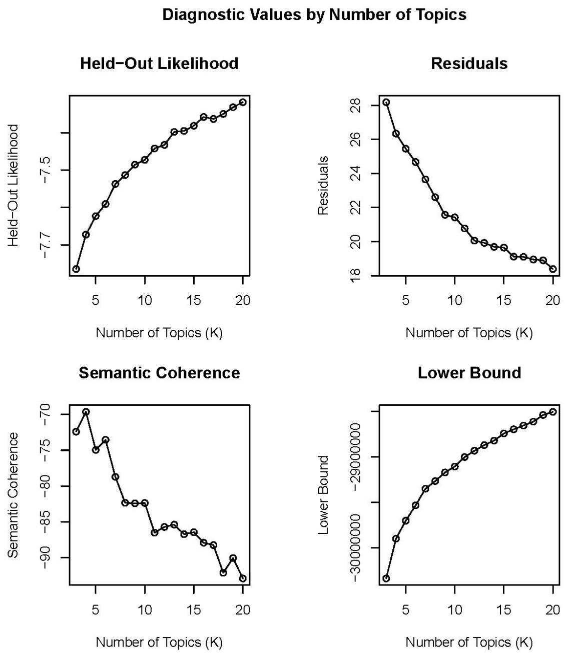

Diagnostic Values for Model 3 (After Policy Change)

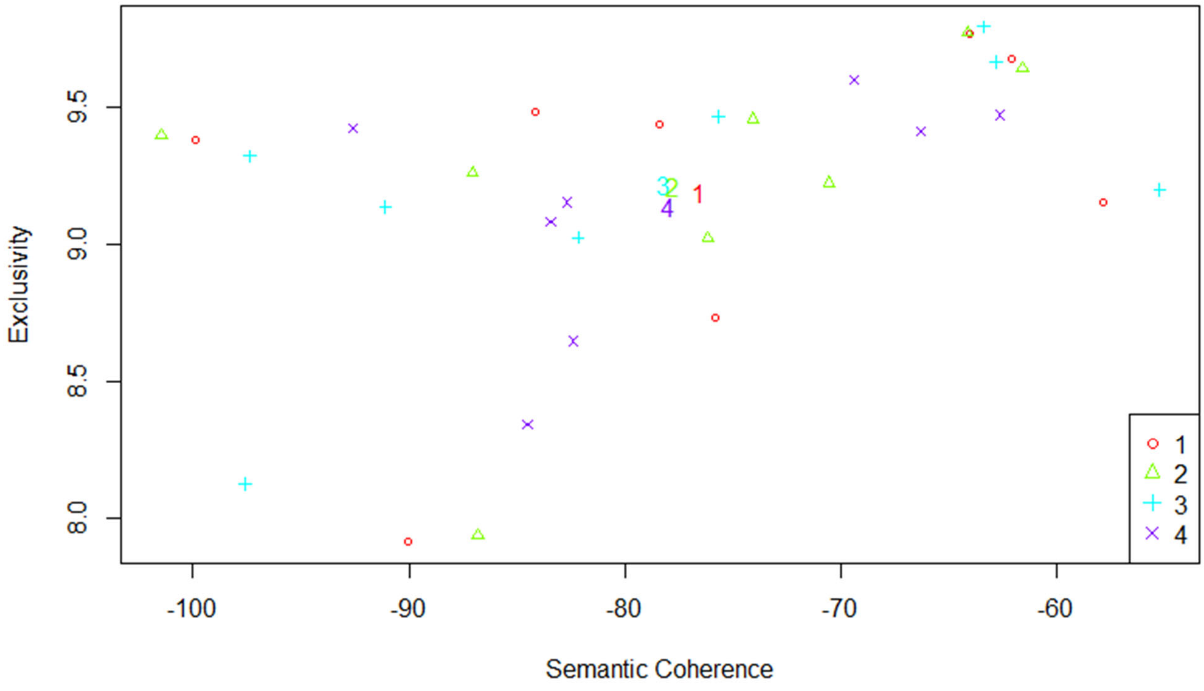

Model 3 Semantic Coherence and Exclusivity Plots.

Colors indicate different model iterations,

Colored numbers indicate averaged values for a color-represented iterations.
