# Supplementary material for: Topic and Trend Analysis of Weibo Discussions About COVID-19 Medications Before and After China’s Exit from the Zero-COVID Policy: Retrospective Infoveillance Study
Source: J Med Internet Res. 2023 Oct 27;25:e48789. doi: 10.2196/48789 (PMC10638631; doi:10.2196/48789)
Supplement: Multimedia Appendix 2 [file jmir_v25i1e48789_app2.pdf]

Multimedia Appendix

Appendix I. Topic Distribution, Proportions within Models, and Keywords.

	Topics	Model 1 Keywords (Whole Timeframe)			Model 2 Keywords (Before Policy Change)			Model 3 Keywords (After Policy Change)		
		Topic % in Model 1	Chinese	English(Trans.)	Topic % in Model 2	Chinese	English(Trans.)	Topic % in Model 3	Chinese	English(Trans.)
Symptoms and Treaments	Purchase and Shortage	22.37%	抢,屯药,没药,囤点,没饭,抢药,买不到,张朝阳,店主,送不来	plunder, stockpile, no drugs, stock up, cannot buy, cannot deliever	26.88%	下架,囤药,顺序,中成药,上呼吸道,解热,李侗,备药,镇痛,处方药	Take down, stockpile, order, upper respitory tract, antipyretic, painkillers, prisciption drugs	23.19%	寄,问问,全羊,没货,几盒,买好,催,买个,给我发,感恩	send, ask, all positive, not in stock, how many, well bought, urge, send to me, thanks
	Symptoms Sharing	18.16%	疼,睡觉,低烧,睡不着,醒来,发冷,被子,烧到,量体温,入睡	pain, sleep, low fever, insomnia, wake, cold, blanket, temperature	30.90%	死,嗓子,难受,怕,睡,疼,睡觉,喉咙,打电话,羊了	die, throat, uncomfortable, afriad, sleep, pain, phone call, positive.	19.88%	疼,睡,头疼,第三天,低烧,第一天,第四天,醒来,睡着,起床	pain, sleep, headache, third day, fever, first day, fourth day, wake, asleep
	Inhalable Vaccines	9.33%	细胞,钟南山,致病,活疫苗,腺病毒,毒力,吸入式,剂灭,康希诺,病死率	cells, Dr. Zhong, inactivated vaccine, adenovirus, inhalable, CanSino	9.97%	调节,搅拌,排气口,进气口,亲和度,直径,粉碎,提取,抑制剂,冷却	adjust, stir, exhaust port, affinity, diameter, pulverize, extract, inhibitor			
	Traditional Chinese Medicine Formulae	19.28%	中成药,解毒,甘草,疏风,疯传,清肺,宣肺,中药方,败毒,解表	Chinese medicine, antidotal, licorice, clear lungs, clear toxins				8.01%	藿香,煮水,清肺,甘草,小儿,宣肺,中药方,正气,解毒,败毒	Agastache rugosa, boil, clear lung, licorice, kids, Chinese medicine formulae, fix Qi, antidote.
	Oral Antivirus Drugs	9.20%	口服药,下架,新冠药,国家药监局,网售,注册,奈玛特韦,辉瑞公司,开售,售价	oral drug, take down, COVID drug, China FDA, Pfizer, Nirmatrelvir, price						
	Specific Populations							15.62%	一图,转存,中青年,心血管,血糖,发热时,抗菌,患儿,传言, 北京协和医院	one picture, save, youth, cardiovascular, blood sugar, fever, antibiotics, kids, rumor, Beijing Xiehe Hospital
	Regulations of Medicines							10.80%	目录,互助,国家药监局,多家,开具,报销,医疗器械,第一版,违法,委托	menu, mutual help, China FDA, prescribe, reimburse, medical equipment, first edition, illegal, delegate.
	Import and Generic Drugs							9.14%	钟南山,美国,院士,香港,仿制,印度,焦点,关税,突变,进化	Dr. Zhong, USA, academician, HongKong, generic drugs, India, focus, tariff, evolve.
	Celebrities' Experiences							8.81%	张兰, 张歆艺,大自然,恐惧,可怕,卖货,张靓颖,剧疼,开窗,乐观	Zhanglan, Zhang Xinyi, nature, afraid, fear, sell, pain, open window, optimism
Governmental Pandemic Control Policy	Global Policy Comparison	9.09%	柬埔寨,共存,李克强,洪森,外国,共同体,牺牲,联合国,全人类,争论	Cambodia, co-existance, Premier Li, Foreign country, human community, UN, humanity, debate						
	Governmental Investigation	4.61%	党中央,滞留,摸清,研究部署,工作汇报,医疗卫生, 推诿, 熔断,中共中央政治局,实际困难	CCP Central Committee, understand, investigate, implement, report, healthcare, fuse mechanism	8.75%	柬埔寨,党中央,接种率,听取,总书记,研究部署,工作汇报,中共中央政治局,主持会议,实际困难	CCP Central Committee, vaccination rate, listen, General Secretary, Investigate, Report, Central PoliBureau, Actual difficulty			
	Pandemic Control Policy	3.92%	暂停营业,绿码,主城区,校外,经营性,经营场所,娱乐场所,影剧院,旅居	temporary closure, green code, main districts, businesses, theatres, hotels	11.05%	暂停,企事业,暂停营业,绿码,实名,主城区,校外,经营性,经营场所,党政机关,临时性	pause, businesses, green code, real ID, main districts	4.55%	优化,机构,落实,基层,医疗卫生,查验,加强免疫,医务人员,强化,剂次	amoliorate, organizations, implement, grass-root, healthcase, immunity, strengthen, doses
COVID-19 Cases Tracking	Cases in Zhengzhou & Dalian	1.63%	外馆,丹尼斯,金水区,南米,甘井子区,西门,旧宫,西米,全日,沈河区,	Waiguan, Denis, Jinshui Dist. Nanmi, West Gate, Jiugong, all day, Shenhe Dist.	4.02%	丹尼斯,甘井子区,沈河区,郑东新区,沙河口区,上街区,郭店,路站,街站,铁西区	Denis, Ganjingzi Dist., Shenhe Dist., Zhengdong Dist., etc.			
	Cases in Wuhan & Beijing	1.48%	现住,金银潭,海珠区,密云,南通市,隔离,确诊,筛查,转运,检测	Lives in, Jinyintan, Haizhu Dist. Miyun Dist. Quarantine, Confirmed, Filter, Transfer, PCR	4.80%	现住,大兴区,密云,昌平区,金盏,北京客隆,旧宫,西城区,顺义区,返京	lives in, Daxing Dist., Miyun Dist., Changping Dist., etc. Return to Beijing			
	Cases in Xi'an and Xinzhou	0.94%	雁塔区,未央区,七一路,莲湖区,长安区,曲江,海州区,长征街,云中路,灞桥区,陵区	Yanta Dist, Weiyang Dist, Qiyi Rd., Lianhu Dist., Chang'an Dist., Qujiang Dist.	2.57%	雁塔区,未央区,七一路,莲湖区,海州区,云中街,灞桥区,西咸新区,三桥,漳浒寨	Yanta Dist., Weiyang Dist., Qiyi Dist., etc.			
	Cases in Dingxiang & Nantong				1.06%	接单,送达,店,定襄,海门,崇川区,南通市,定襄县,江岸区,晋昌店	take order, delivered, shop, Dingxiang, Haimen, Chongchuan Dist., Nantong City., etc.			
